# CrossFit^®^: Injury prevalence and main risk factors

**DOI:** 10.6061/clinics/2019/e1402

**Published:** 2019-11-19

**Authors:** Taline Santos da Costa, Clayder Tobias Navega Louzada, Gustavo Kenzo Miyashita, Paulo Henrique Jeronimo da Silva, Heloisa Yumi Fujiya Sungaila, Paulo Henrique Schmidt Lara, Alberto de Castro Pochini, Benno Ejnisman, Moisés Cohen, Gustavo Gonçalves Arliani

**Affiliations:** Centro de Traumatologia do Esporte (CETE), Departamento de Ortopedia e Traumatologia, Universidade Federal de Sao Paulo, Sao Paulo, SP, BR

**Keywords:** Injuries, Prevalence, Sports

## Abstract

**OBJECTIVES:**

This study sought to determine prevalences of injuries associated with CrossFit^®^ training and assess profiles of these injuries and the affected athletes.

**METHODS:**

Data were collected over a 12-month period using a questionnaire administered directly to practitioners at CrossFit^®^-affiliated fitness centers in the state of São Paulo, Brazil. Of the 414 participants, 157 (37.9%) participants reported having suffered an injury while practicing CrossFit^®^.

**RESULTS:**

The injury rate was 3.24 injuries per 1,000 hours of training. The probability of injury for athletes who had practiced CrossFit^®^ for longer than 12 months was 82.2%, which was higher than the corresponding probability for beginner athletes. The probability of injury was 5-fold higher among competitive-level athletes than that among less-experienced athletes. No evidence of an association between the occurrence of injuries during CrossFit^®^ practice and any of the following athlete characteristics was found: age, gender, practice of other sports, weight, and height. The incidence of injuries in this sports modality was similar to that in other modalities, including Olympic weight lifting (OWL), basic weight lifting, and artistic gymnastics.

**CONCLUSION:**

CrossFit^®^ appears to be a training program that is suitable for different age groups when performed in a safe environment and with assistance from qualified professionals.

## INTRODUCTION

CrossFit^®^ is a training program designed to promote general physical conditioning and improve health based on the principles of exercise variability, high-intensity training, and functional movements (1). The objective of the program is to improve the physical capacities of the human body, including cardiorespiratory resistance, strength, vigor, power, speed, coordination, flexibility, agility, balance, and precision (2).

Training sessions begin with stretching and warm-up, followed by a combination of strength-building exercises or specific skills. Subsequently, the “workout of the day” (WOD) is performed. Exercises vary from Olympic weight lifting (OWL), squats, snatches, throws, aerobic exercises (such as rowing, running, and cycling), and gymnastic movements (such as hand stands, planks, rings, and bars) (3). Individuals of different levels of proficiency can exercise in the same training sessions because the loads/movements are adjusted on an individual basis considering the level of skill and conditioning of each practitioner (4).

CrossFit^®^ has gained substantial popularity since it began in the year 2000. There are more than 13,000 CrossFit^®^-affiliated centers worldwide and approximately 683 certified and registered training centers in Brazil. Of these, 490 are located in São Paulo state and are attended by approximately 40,000 practitioners (1,4). However, the safety of this practice as a sport modality has received criticism because it involves technical high-intensity exercises. To date, the data from the literature are contradictory, and only a few studies determined the incidence and prevalence of injuries in this modality.

The objective of this study was to determine the prevalence of injuries and assess the profile of athletes and injuries associated with CrossFit^®^ training.

## MATERIALS AND METHODS

This observational, descriptive, cross-sectional, epidemiological study evaluated injuries associated with CrossFit^®^ practice and their relationship with demographic variables and the adopted training regimen. The data were collected from April 2015 to April 2016 at several CrossFit^®^-affiliated fitness centers in the state of São Paulo using a printed questionnaire applied directly to the practitioners of this modality.

The questionnaire was composed of 18 questions related to demographic data and the training regimen (Appendix).

The questionnaires were applied by the study authors in CrossFit^®^-affiliated centers, with the consent of the directors of these centers and the practitioners (or their parents or legal guardians). The researcher was present during the completion of the questionnaire to answer any possible questions

The adopted concept of injury was that any sensation, pain, or injury during CrossFit^®^ training that resulted in one or more of the following outcomes was considered to be a physical injury:

- Complete withdrawal from CrossFit^®^ training or other routine physical activity for a period longer than 1 week;
- Modification of normal training activity in duration, intensity, or modality for a period longer than 2 weeks; or
- Any physical complaint severe enough to make the practitioner seek medical help.

This concept of injury was the same as that used in prior studies (3,4).

The inclusion criteria were athletes who practiced CrossFit^®^ in affiliated academies in the state of São Paulo, were age 16 years or older, had at least 6 months of CrossFit^®^ practice, gave consent to participate in the study, and completed the entire questionnaire.

The numerical variables were described by the mean and standard deviation (SD) or median and quartiles (Q1: first quartile, and Q3: third quartile), and minimum and maximum values, and the categorical variables were described by the absolute and relative frequencies.

The association between injury and the athletes’ characteristics and sports practice characteristics was evaluated by fitting logistic regression and simple linear regression models, and the results are presented as odds ratios (ORs) and 95% confidence intervals (95% CIs).

Statistical analysis was conducted using SPSS^®^ software version 19 and a significance level of 0.05.

The study was approved by the Research Ethics Committee of the Federal University of São Paulo.

## RESULTS

A total of 477 questionnaires were collected, of which 63 participants (13%) did not meet the inclusion criteria, and thus, 414 practitioners were included in the study. The characteristics of the participants and their level of physical exercise are described in Table 1.

A total of 79.2% of the participants reported performing other sports modalities other than CrossFit^®^, and the most common modalities were running, bodybuilding, cycling, and martial arts.

Of the 414 participants, 157 (37.9%) reported having suffered injuries during CrossFit^®^ practice. The rate of injuries per 1,000 hours of training was 3.24.

**Figure 1:**
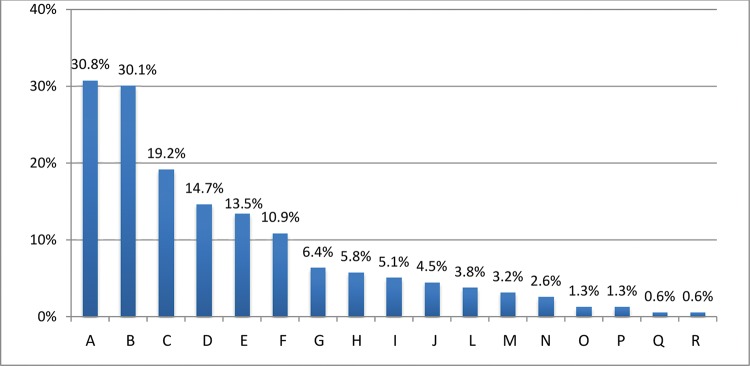
Body regions affected by injuries. A=Shoulder, B=Low lumbar spine, C=Leg, D=Wrist, E=Knee, F=Hand/finger, G=Ankle, H=Thigh, I=Arm, =Cervical spine, L=Foot, N=Head/face, O=Abdomen, P=Elbow, Q=Sternum/ribs/high thoracic spine, R=Forearm.

The most affected body sites were the shoulder and lumbar spine (Figure 1). The percentage of injuries that required surgical treatment was low, representing 2.6% of the total (n=4), which was in agreement with the relative frequency distribution based on the severity scale: mild to moderate injuries represented 89.1% (n=139) of the cases whereas severe and very severe injuries represented only 10.9% (n=17) of the cases.

No evidence of an association between injuries during CrossFit^®^ practice and the following variables was found: athlete’s age, gender, practice of other sports, weight, or height. However, the presence of previous injuries and the period of CrossFit^®^ practice were significant risk factors. One month in the period of CrossFit^®^ practice increased the probability of injury by 3.2. The probability of injury in athletes with a period of practice longer than 12 months was 82.2% higher than for athletes with a period of practice of up to 12 months.

Moreover, there was an association between the presence of injuries and the level of proficiency of the athlete (*p*<0.001). The probability of injuries in competitive-level athletes was 5-fold higher than for beginner athletes, and the probability of injuries in recreational-level athletes was approximately 2-fold higher than for beginners.

The most common injuries were muscle strains (41.0%), overload injuries (26.2%), and contusions (17.3%). Fractures and dislocations accounted for 5.6% of the cases. Furthermore, there was no difference in the injury rate between the groups that practiced sports other than CrossFit^®^ and the athletes that did not (*p*=0.730).

Table 2 shows the results of the logistic regression models used to evaluate the association between the presence of injury and the characteristics of the athletes and their sports practice.

## DISCUSSION

The incidence of CrossFit^®^ injuries in this study was 3.24 per 1,000 hours of exercise, and the prevalence of injuries was approximately 36%. A recent survey conducted in Brazil (4) reported a similar rate (31%, 176 of 566 practitioners). The incidence rate reported by Hak et al. (5) (3.1) was similar to the incidence rate of this study and the study by Montalvo et al. (6) (2.3). However, the prevalence rate found in this study was much lower than the prevalence rate reported by Hak et al. (5) (73.5%) among 132 CrossFit^®^ practitioners (6).

These incidence rates are similar to the rates found for other modalities, including OWL, basic weightlifting, and artistic gymnastics (7,8). These results suggest that body movements during CrossFit^®^ practice are the primary contributors to injuries. In addition, the incidence of injury was lower than the average rate reported among recreational street runners (7.7 per 1,000 hours) (9), which is a widely disseminated sport modality among athletes.

With respect to the location of the injuries, the shoulder and low lumbar spine were the most affected sites. These findings agree with the findings of other studies, which indicated that these two regions were the most affected in OWL and Olympic gymnastics, respectively (3,5), and indicate the importance of adopting specific preventive measures to reduce the incidence of injuries. In addition, CrossFit^®^ practitioners need to correctly execute movements with weights to avoid injuries in these risk areas.

With regard to the injury severity scale, there was a predominance of mild to moderate injuries (89.1%). In addition, only 2.6% of the injuries required surgical intervention, which was lower than the rate found by Weisenthal et al. (3) (7%). However, those authors also found that the severity of most injuries (72/89; *p*<0.001) was mild, as observed in the present study.

There was a significant association between the presence of injuries and the period of CrossFit^®^ practice. The likelihood of injuries in athletes with a period of practice longer than one year was 82.2%, which was higher than the rate for less experienced athletes. Therefore, it can be inferred that competitive-level athletes tend to suffer more injuries than recreational athletes and beginners.

This hypothesis was confirmed in our study because we found that the probability of injuries among competitive-level athletes was approximately 5 times higher than among beginners. One possible explanation is that competitors need to achieve a higher level of technical proficiency and thus require a longer period of practice. As discussed earlier, the greater the exposure to physical activity, the higher the probability of the occurrence of injuries. Moreover, competitions pose a risk of injury because of the need to overcome limits. These results are similar to the results found by Montalvo et al. (6) involving 191 practitioners, wherein competitors were more susceptible to injuries (40% *vs*. 19%, *p*=0.002) and had a longer period of CrossFit^®^ practice than other athletes (2.7±1.8 *vs*. 1.8±1.5, *p*=0.001).

There was no significant difference in the rate of injury among male and female practitioners. In addition, there was no significant difference in the mean age of practitioners with different types of injuries. These results suggest that CrossFit^®^ is suitable for different age groups when performed in a safe environment and with the help of qualified professionals (1). In this respect, experienced physical trainers should assist practitioners in adapting to the exercise program, and the training program should be designed on an individual basis considering the level of conditioning and other physical limitations of each practitioner. The observation that the shoulder and lumbar spine are common sites of injuries indicates that physical trainers should devote particular attention to these locations, focusing on strategies to minimize injury risk.

An important consideration is that 79.2% of the participants reported performing other sports modalities other than CrossFit^®^; thus, there was a risk of bias because it was impossible to assume that all injuries were caused by a single sports modality.

The limitation of the present study was the use of a questionnaire with retrospective evaluation of the injuries. In addition, the topographic diagnosis was not specific because it was not performed by a physician and thus depended on the practitioner’s interpretation of the injury. However, the application of the questionnaire in person by qualified physicians allowed a high adherence to the survey, the clarification of any questions from the participants, and consequently the collection of a large population sample. Although the prevalence of injuries was similar to the prevalence in other studies, more prospective studies are necessary to expand the knowledge on injuries associated with CrossFit^®^ practice and the strategies to prevent these injuries.

## CONCLUSION

The prevalence of injuries among CrossFit^®^ practitioners was 36%, and the rate of injuries per 1,000 hours of training was 3.2. The probability of injury increased as the period of practice and level of proficiency of the athletes increased. Most injuries were of mild to moderate severity and occurred mainly in the shoulder and lumbar spine.

### AUTHOR CONTRIBUTIONS

Costa TS, Louzada CTN, Miyashita GK, Silva PHJ and Sungaila HYF were responsible for the manuscript writing, statistical analysis, intellectual concept and implementation of the entire research project. Lara PHS, Pochini AC, Ejnisman B, Cohen M and Arliani GG were responsible for the manuscript writing, review and intellectual concept.

## APPENDIX

CrossFit^®^ Questionnaire

- Name:_____________________ Date of birth:______________________
- Phone:_____________________ Email:____________________________
- Gender: ( ) Male ( ) Female
- Profession:____________________________________________________
- Other sport: ( ) Soccer ( ) Volleyball ( ) Basketball ( ) Handball ( ) Running ( ) Walking
- Other:________________________________________________________
- Level of sport: ( ) Professional ( ) Amateur ( ) Recreational
- Weight:________kg Height:________m
- Dominant member in the superior extremity: ( ) Right ( ) Left
- Dominant member in the inferior extremity: ( ) Right ( ) Left
- How many years of CrossFit practice?___________________________
- How many hours per week you practice CrossFit: ( ) 2 ( ) 3 ( ) 4 ( ) 5 ( ) 6 ( ) 7
- Have you ever had a injury related to the CrossFit? ( ) Yes ( ) No
- How many injuries? ( ) 1 ( ) 2 ( ) 3 ( ) 4 ( ) 5 ( ) 6 ( ) 7 ( ) >7
- Date of the injuries:____________________________________________
- Injury caused by: ( ) Trauma ( ) Overuse
- Part of the body where the injury occurred: ( ) Head/Face ( ) Neck/Cervical spine ( ) Ribs ( ) Abdomen ( ) Lumbar spine ( ) Shoulder ( ) Arm ( ) Elbow ( ) Forearm ( ) Wrist ( ) Hand ( ) Hip ( ) Thigh ( ) Knee ( ) Leg ( ) Ankle ( ) Foot
- Side of the injury: ( ) Right ( ) Left ( ) Not applicable
- Type of injury: ( ) Muscular stretching ( ) Sprain. Which?________ ( ) Contusion ( ) Dislocation. Which articulation?__________ ( ) Wound. Where?__________ ( ) Fracture. Which?___________ ( ) Others:______________ ( ) Overuse (tendinopathy, stress fracture)
- Exams: ( ) Radiography ( ) Ultrassonography ( ) Tomography ( ) Magnetic Ressonance ( ) Others:______________
- Medicament: ( ) Yes ( ) No. Which?______________________________
- If yes, was it with medical prescription? ( ) Yes ( ) No
- Did the injury demand medical support? ( ) Yes ( ) No
- Did the injury demand hospitalization? ( ) Yes ( ) No
- Did the injury demand surgery? ( ) Yes ( ) No. If yes, which?__________
- Days of withdrawal from sport:__________________________________
- Injury severity: ( ) Mild ( ) Minor ( ) Moderate ( ) Major ( ) Severe
- Did you do physiotherapy? ( ) Yes ( ) No

## Figures and Tables

**Table 1:** Personal and sports practice data of CrossFit^®^ practitioners (n=414).

Age (years)	
Mean (SD)	31.0 (6.6)
Minimum-maximum	16-60
Number of athletes	409
Sex	
Male	243 (58.7%)
Female	171 (41.3%)
Practice of other sports	
No	76 (20.8%)
Yes	290 (79.2%)
Number of athletes	366
Weight (kg)	
Mean (SD)	73.7 (13.6)
Minimum-maximum	48-130
Number of athletes	413
Height (m)	
Mean (SD)	1.72 (0.09)
Minimum-maximum	1.43-1.97
Number of athletes	413
BMI (kg/m^2^)	
Mean (SD)	24.8 (2.9)
Minimum-maximum	18.7-36.4
Number of athletes	413
Period of CrossFit^®^ practice (months)	
Median (Q1; Q3)	12 (6; 24)
Minimum-maximum	6-60
Number of athletes	411
Hours of CrossFit^®^ practice per week	
Median (Q1; Q3)	5 (4; 6)
Minimum-maximum	2-12
Number of athletes	413

**Table 2:** Association between the presence of injury and the characteristics of athletes and their sports practice.

Variable	Injury during CrossFit® practice		OR (95% CI)	*p*-value
	No	Yes		
Age				
Mean (SD)	30.9 (6.7)	31.2 (6.5)	1.007 (0.977, 1.038)	0.659
Sex				
Male (n=243)	154 (63.4%)	89 (36.6%)	1.000	
Female (n=171)	103 (60.2%)	68 (39.8%)	1.142 (0.764, 1.708)	0.517
Practice of other sports				
No (n=76)	45 (59.2%)	31 (40.8%)	1.000	
Yes (n=290)	177 (61.0%)	113 (39.0%)	1.161 (0.750; 1.797)	0.504
Weight				
Mean (SD)	73.7 (14.0)	73.7 (12.9)	1,000 (0.986; 1.015)	0.985
Height				
Mean (SD)	1.72 (0.09)	1.71 (0.09)	0.401 (0.047; 3.448)	0.405
BMI				
Mean (SD)	24.7 (3.0)	24.9 (2.7)	1.032 (0.963; 1.106)	0.367
Period of practice				
Median (Q1; Q3)	10 (4; 20)	13 (9; 24)	1.032 (1.013; 1.051)	0.001
Period of practice				
≤12 months (n=239)	163 (68.2%)	76 (31.8%)	1.000	
>12 months (n=172)	93 (54.1%)	79 (45.9%)	1.822 (1.215; 2.732)	0.004
Level of proficiency				
Competitive (n=63)	25 (39.7%)	38 (60.3%)	5.262 (2.700; 10.254)	<0.001
Recreational (n=232)	140 (60.3%)	92 (39.7%)	2.275 (1.367; 3.786)	0.002
Beginner (n=116)	90 (77.6%)	26 (22.4%)	1.000	

OR: Odds ratio; 95% CI: 95% confidence interval; SD: standard deviation; Q1: first quartile; Q3: third quartile.

The OR is 1.000 for reference categories.
